# Branched-Chain Amino Acid Levels Are Related with Surrogates of Disturbed Lipid Metabolism among Older Men

**DOI:** 10.3389/fmed.2016.00057

**Published:** 2016-11-25

**Authors:** Urho M. Kujala, Markku Peltonen, Merja K. Laine, Jaakko Kaprio, Olli J. Heinonen, Jouko Sundvall, Johan G. Eriksson, Antti Jula, Seppo Sarna, Heikki Kainulainen

**Affiliations:** ^1^Department of Health Sciences, University of Jyväskylä, Jyväskylä, Finland; ^2^Diabetes Prevention Unit, Department of Chronic Disease Prevention, Division of Welfare and Health Promotion, National Institute for Health and Welfare, Helsinki, Finland; ^3^Department of General Practice and Primary Health Care, Helsinki University Hospital, University of Helsinki, Helsinki, Finland; ^4^Vantaa Health Center, Vantaa, Finland; ^5^Department of Public Health, University of Helsinki, Helsinki, Finland; ^6^Department of Health, National Institute for Health and Welfare, Helsinki, Finland; ^7^Institute for Molecular Medicine, University of Helsinki, Helsinki, Finland; ^8^Department of Health and Physical Activity, Paavo Nurmi Centre, University of Turku, Turku, Finland; ^9^Genomics and Biomarkers Unit, Department of Health, National Institute for Health and Welfare, Helsinki, Finland; ^10^Folkhälsan Research Center, Helsinki, Finland; ^11^Population Research Unit, Department of Chronic Disease Prevention, National Institute for Health and Welfare, Turku, Finland; ^12^Department of Biology of Physical Activity, University of Jyväskylä, Jyväskylä, Finland

**Keywords:** branched-chain amino acids, metabolic disease, tricarboxylic acid cycle, lipid oxidation, mitochondria, energy metabolism

## Abstract

**Aims/hypothesis:**

Existing studies suggest that decreased branched-chain amino acid (BCAA) catabolism and thus elevated levels in blood are associated with metabolic disturbances. Based on such information, we have developed a hypothesis how BCAA degradation mechanistically connects to tricarboxylic acid cycle, intramyocellular lipid storage, and oxidation, thus allowing more efficient mitochondrial energy production from lipids as well as providing better metabolic health. We analyzed whether data from aged Finnish men are in line with our mechanistic hypothesis linking BCAA catabolism and metabolic disturbances.

**Methods:**

Older Finnish men enriched with individuals having been athletes in young adulthood (*n* = 593; mean age 72.6 ± 5.9 years) responded to questionnaires, participated in a clinical examination including assessment of body composition with bioimpedance and gave fasting blood samples for various analytes as well as participated in a 2-h 75 g oral glucose tolerance test. Metabolomics measurements from serum included BCAAs (isoleucine, leucine, and valine).

**Results:**

Out of the 593 participants, 59 had previously known type 2 diabetes, further 67 had screen-detected type 2 diabetes, 127 impaired glucose tolerance, and 125 impaired fasting glucose, while 214 had normal glucose regulation and one had missing glucose tolerance information. There were group differences in all of the BCAA concentrations (*p* ≤ 0.005 for all BCAAs), such that those with normal glucose tolerance had the lowest and those with diabetes mellitus had the highest BCAA concentrations. All BCAA levels correlated positively with body fat percentage (*r* = 0.29–0.34, *p* < 0.0001 for all). Expected associations with high BCAA concentrations and unfavorable metabolic profile indicators from metabolomics analysis were found. Except for glucose concentrations, the associations were stronger with isoleucine and leucine than with valine.

**Conclusion/interpretation:**

The findings provided further support for our hypothesis by strengthening the idea that the efficiency of BCAA catabolism may be mechanistically involved in the regulation of fat oxidation, thus affecting the levels of metabolic disease risk factors.

## Introduction

High serum branched-chain amino acid (BCAA; isoleucine, leucine, and valine) concentrations have been shown to be predictors or markers of insulin resistance (1), obesity (2, 3), diabetes (4, 5), and atherogenetic dyslipidemia (6, 7) and response to a test meal (8). A 12-year prospective follow-up study of initially normoglycemic individuals showed an association between high serum BCAA levels and incidence of type 2 diabetes (4). In our studies, the metabolomics of twin pairs discordant for leisure-time physical activity for 30 years revealed lower concentrations of serum BCAAs, especially isoleucine, in the active vs. inactive co-twin (9). Serum isoleucine was confirmed to be low in physically active persons in three different population-based cohorts (9). Similarly, the transcriptomics of skeletal muscle and adipose tissue yielded upregulated mRNA expression signature of BCAA catabolism and fatty acid metabolism in active compared to inactive co-twins (10). Animal studies support the findings in humans (11, 12). BCAAs are oxidized and used for energy production during exercise. Interestingly, the prevention of the first step of BCAA catabolism by deleting the *Bcat2* gene encoding BCAA aminotransferase, the first enzyme in BCAA catabolism, renders mice exercise intolerant, affirming that BCAA catabolism is necessary for exercise performance (13). Skeletal muscle has the highest capacity not only to catabolize BCAAs in humans but also adipose tissue, brain, and liver play important roles (14).

**Figure 1:**
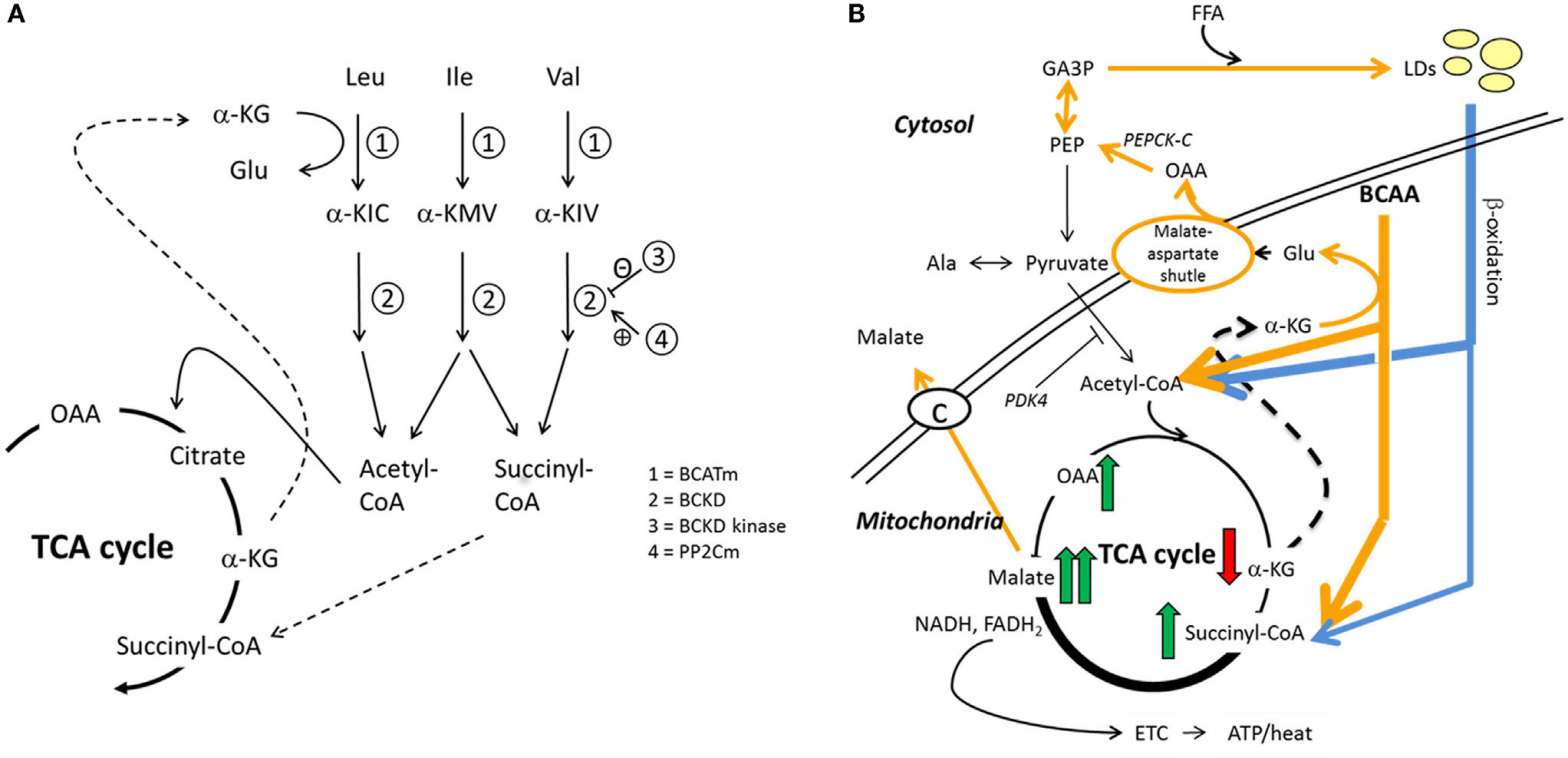
**Hypothesized interplay between skeletal muscle BCAA catabolism and fatty acid metabolism**. We recently proposed how BCAA degradation mechanistically connects to lipid storage and oxidation in skeletal muscle (15). **(A)** In mitochondria, the first reaction of the BCAA catabolism is the transfer of amino group from BCAA to a tricarboxylic acid (TCA) cycle intermediate α-ketoglutarate (α-KG) to form glutamate (Glu) in a reaction catalyzed by mitochondrial branched-chain aminotransferase (BCATm). Deaminated BCAAs are further decarboxylated and processed to supply acetyl-CoA and succinyl-CoA for the TCA cycle. **(B)** In this model, cytosolic oxaloacetate is further metabolized to phosphoenolpyruvate for glyceroneogenesis, which is required in skeletal muscles for the storage of fatty acids in lipid droplets (LDs). Similarly, β-oxidation of fatty acids provides acetyl-CoA and also succinyl-CoA. It is worth noting that during moderate-intensity exercise acetyl-CoA supply from pyruvate is inhibited by PDK4-catalyzed phosphorylation of the pyruvate dehydrogenase complex, thus favoring β-oxidation. During exercise, the muscle concentration of α-ketoglutarate decreases and the concentrations of the other TCA metabolites increase, the concentration of malate being the highest. In the presented model, cytosolic malate, which is required for the functioning of the malate–aspartate shuttle, is transported from the mitochondria to cytosol *via* the dicarboxylate carrier. Both TCA cycle and β-oxidation provide NADH and/or NADPH to electron transport chain for cellular energy production (ATP/heat). Shortly, we suggested that during increased demand for BCAA catabolism, e.g., during exercise due to increased energy-yielding substrate requirement, transamination of BCAAs is essential for cytosolic oxaloacetate formation. Ala, alanine; α-KIC, α-ketoisocaproate; α-KIV, α-ketoisovalerate; α-KMV, α-keto-β-methylvalerate; BCKD, branched-chain α-ketoacid dehydrogenase; BCKD kinase, branched-chain α-ketoacid dehydrogenase kinase; ETC, electron transport chain; FFA, free fatty acid; GA3P, glyceraldehyde-3-phosphate; Ile, isoleucine; Leu, leucine; OAA, oxaloacetate; PDK4, pyruvate dehydrogenase kinase-4; PEP, phosphoenolpyruvate; PEPCK-C, cytosolic phosphoenolpyruvate carboxykinase; PP2Cm, BCKD phosphatase; Val, valine. Reproduced by the permission provided by Wolters Kluwer Health, Inc. from the article: Kainulainen et al. (15).

In sum, the existing metabolomic and transcriptomic studies suggest that decreased BCAA catabolism, and thus elevated levels in blood are associated with low physical activity, increased adiposity, and other risk factors for metabolic diseases. We have developed a mechanistic hypothesis (BCAA–FatOx hypothesis) that intertwines BCAA catabolism with the tricarboxylic acid (TCA) cycle and lipid metabolism allowing better lipid oxidation and better metabolic health (Figure 1) (15), thus completely explaining the mechanistic background of these associations. The aim of the current study was to test whether data from elderly Finnish men are in line with this mechanistic hypothesis. In more detail, we tested whether indicators of impaired oxidative fat metabolism selected on the basis of earlier reports (9, 15–21) (impaired glucose regulation, high body fat percentage, impaired liver metabolism, low leisure-time physical activity, and accumulation of indicators of low fat oxidation in lipoprotein sub-fractions) are associated with increased serum BCAA concentrations.

## Materials and Methods

### Participants and Study Design

The participants were members of the cohort of male former Finnish elite athletes and their controls (22, 23), which means that the cohort was enriched with individuals having a documented history of an extended period of high physical activity level. The original cohort (*n* = 4136) consisted of male former elite athletes who represented Finland between the years 1920 and 1965 at least once in international competitions (Olympic Games, World or European Championships, or other intercountry competitions) and their age- and area-matched controls. The controls were classified as healthy at the medical examination, which all Finnish men undergo at age 20 years as part of the national military service. By the year 2008, 2702 (65.3%) of the cohort members had died.

Of the surviving cohort members, 1183 were invited to a clinical study in 2008 (24). The invitation was sent to all surviving cohort members who had responded at least once to the previous questionnaires sent in 1985, 1995, or 2001. Of those invited, 599 participated in the physical examination and completed the questionnaires. Of the participants, 594 provided fasting blood samples. One of these had type 1 diabetes and was excluded from the study, thus 593 comprised the target group of this paper (Table 1).

**Table 1:** **Participant characteristics according to glucose tolerance**.

	Normal (*n* = 214)	IGT or IFG (*n* = 252)	T2D or ST2D (*n* = 126)	*p*-Value^†^

	Mean ± SD			
Age (years)	71.6 ± 6.1	72.7 ± 5.9	72.9 ± 5.7	0.065
Height (cm)	176 ± 7	175 ± 8*	174 ± 8**	0.010
Weight (kg)	79.7 ± 10.8	81.6 ± 13.2	85.7 ± 16.6**	0.004
Body mass index (kg/m^2^)	25.6 ± 2.7	26.6 ± 3.5**	28.2 ± 4.7***	<0.001
Body fat (%)	23.7 ± 4.9	25.3 ± 5.7**	27.4 ± 6.4***	<0.001
Leisure-time physical activity volume (MET-h/week)	32.3 ± 29.7	28.3 ± 26.6*	18.8 ± 19.3***	<0.001
Alanine aminotransferase (U/L)	23.7 ± 9.9	26.1 ± 11.2*	30.7 ± 21.0**	0.013
γ-glutamyltransferase (U/L)	32.1 ± 19.4	36.5 ± 30.8	53.1 ± 56.2***	<0.001
Glucose (mmol/L)	4.31 ± 0.24	4.76 ± 0.32***	5.60 ± 1.42***	<0.001
Serum total triglycerides (mmol/L)	1.03 ± 0.33	1.09 ± 0.39	1.17 ± 0.59	0.064
Total fatty acids (mmol/L)	9.91 ± 1.59	9.92 ± 1.81	10.03 ± 2.50	0.934
Total lipids in chylomicrons and extremely large VLDL (μmol/L)	17 ± 13	19 ± 15	23 ± 34	0.168
Total lipids in very large VLDL (μmol/L)	38 ± 36	45 ± 40*	55 ± 74*	0.029
Total lipids in large VLDL (μmol/L)	162 ± 123	183 ± 141	214 ± 212	0.065
Mean diameter for VLDL particles (nm)	35.96 ± 1.10	36.13 ± 1.11	36.32 ± 1.30**	0.017
Mean diameter for HDL particles (nm)	9.97 ± 0.24	9.94 ± 0.25	9.90 ± 0.24**	0.007
Ratio of apolipoprotein B to apolipoprotein A-I	0.565 ± 0.109	0.566 ± 0.118	0.557 ± 0.130	0.645
Glycoprotein acetyls, mainly α1-acid glycoprotein (mmol/L)	1.39 ± 0.15	1.43 ± 0.16**	1.48 ± 0.20***	<0.001
Isoleucine (μmol/L)	53 ± 10	55 ± 11	59 ± 15***	0.001
Leucine (μmol/L)	78 ± 12	80 ± 14	87 ± 22***	<0.001
Valine (μmol/L)	179 ± 29	185 ± 29*	196 ± 45***	<0.001

*Data are mean ± SD*.

*^†^p for group differences by Kruskal–Wallis test*.

Statistical difference compared to normal glucose group using Dunn procedure; *p < 0.05; **p < 0.01; ***p < 0.001.

The ethics committee of the Hospital District of Helsinki and Uusimaa approved the study, and all subjects have provided written informed consent.

### Measurements and Definitions

Trained study nurses performed the physical examinations including assessment of body weight, height, body composition, and blood sampling in field survey laboratories around Finland.

Body composition was determined in light indoor clothing and without shoes and socks by a bioimpedance body composition device (InBody 3.0, Biospace, Seoul, South Korea): fat-free mass (FFM) to an accuracy of 0.1 kg and fat mass to an accuracy of 0.1 kg. Fat percent was calculated as fat mass divided by body weight and converted to percent. The body composition device measured body weight to an accuracy of 0.1 kg. If the participant had a pacemaker causing an exclusion from bioimpedance body composition analysis (*n* = 14), weight was measured by a digital scale with the same accuracy. Height was measured without shoes on with a measuring tape against the wall to an accuracy of 0.1 cm. Body mass index (BMI) was calculated as body weight divided by the square of the body height (kilograms per square meter).

Venous blood samples were taken after 10 h fasting in a sitting position with a light stasis into a serum gel tube (Venosafe, Terumo Europe) for metabolomics, lipid, and liver enzyme assays into a serum tube (Venosafe) for insulin assay and into a fluoride–citrate tube (Venosafe) for glucose assay. The venous blood samples were centrifuged at the field survey sites. The plasma and sera were frozen immediately after separating and transferred in dry ice to the laboratory once a week for analyses. Lipids, alanine aminotransferase (Alat), aspartate aminotransferase (AST), γ-glutamyl transferase (Gt), insulin, and glucose measurements were performed on a clinical chemistry analyzer Architect ci8200 (Abbott Laboratories, Abbott Park, IL, USA) at the laboratory of National Institute for Health and Welfare (Helsinki, Finland). The following methods were used: enzymatic assay (Abbott Laboratories) for measuring serum total cholesterol, homogenous assay (Abbott Laboratories) for direct measurement of serum high density lipoprotein (HDL) cholesterol, enzymatic glycerol phosphate oxidase assay (Abbott Laboratories) for measuring serum triglycerides, International Federation of Clinical Chemistry (IFCC) method (Abbott Laboratories) for measuring ALT and AST, kinetic method (Abbott Laboratories) for measuring GT, chemiluminescent microparticle immunoassay (CMIA, Abbott Laboratories) for measuring insulin and enzymatic hexokinase assay (Abbott Laboratories) for plasma glucose. Low density lipoprotein (LDL) cholesterol was calculated by the Friedewald formula (25). For standardizing measurements, the laboratory has taken part in Lipid Standardization Program organized by CDC (Atlanta, GA, USA) and External Quality Assessment Schemes organized by Labquality (Helsinki, Finland). During the course of the study, the coefficient of variation of control samples (CV) (mean ± SD) was 0.9% ± 0.2 for total cholesterol, 1.9% ± 0.5 for HDL-cholesterol, 1.9% ± 0.5 for triglycerides, 4.9% ± 5.0 for ALT, 1.6% ± 1.0 for AST, 1.6% ± 0.4 for GT, 1.9% ± 0.4 for insulin, and 1.8% ± 0.3 for glucose.

Participating men without a history of diabetes had a standard 2 h 75 g OGTT. A blood sample was drawn 2 h after the ingestion of the 300 mL solution, containing 75 g anhydrous glucose and 0.8 g citric acid. In this study, a definition of type 2 diabetes impaired fasting glucose (IFG) and impaired glucose tolerance (IGT) was done according to WHO criteria from year 1999 (26). If the participant had both IFG and IGT, he was defined to have IGT. One participant was unclassified due to missing data from the OGTT.

Metabolic syndrome was defined according to criteria of the International Diabetes Federation: waist circumference >94 cm plus any two of the following factors: (i) TG >1.7 mmol/L or specific treatment for this, (ii) HDL <1.03 mmol/L or specific treatment for this, (iii) systolic BP ≥130 or diastolic BP ≥85 mm Hg or treatment of previously diagnosed hypertension, (iv) fasting plasma glucose ≥5.6 mmol/L or previously diagnosed type 2 diabetes (27). Lipid values measured at laboratory in National Institute for Health and Welfare were used for metabolic syndrome determination.

### Metabolomics Analysis

Serum samples were kept at −80°C until analyzed with a NMR metabolomics platform, which provides quantitative information on lipoprotein subclass lipid distribution and particle concentrations, serum lipids including fatty acids, and low-molecular-weight metabolites such as amino acids (9, 16, 28). Updated information on the current version of the metabolomics platform used is described at http://computationalmedicine.fi/platform. From these data, selected metabolic measures (Table 1) reflecting disturbed oxidative lipid metabolism together with the BCAA’s were included in the present analysis. Specific lipid values from metabolomics platform were used to study their associations with BCAA levels.

### Statistical Analysis

Data have been reported as means ± SD. Group differences in BCAA levels were tested using Mann–Whitney *U*-test or Kruskal–Wallis test with *post hoc* comparisons using Dunn procedure. Spearman correlations with 95% confidence intervals between BCAAs and other serum indicators of disturbed oxidative lipid metabolism were calculated with bootstrapping (1000 samples). Further, age-, physical activity-, and body fat percentage-adjusted correlations were calculated utilizing the partial correlation procedure with bootstrapping. All analyses were carried out using the IBM SPSS version 21.0 (IBM Ltd., Armonk, NY, USA). *p* Values <0.05 were considered statistically significant. As we studied the association between disturbed metabolism and BCAA values, as age adjustment influenced our results only minimally and as in our material the former endurance athletes were older than other participants and they are known to have better metabolic health than other participants (24) we give our results on our aged study group without age-adjustment. Graphical network analysis of metabolomics variables, physical activity measures (MET), body fat%, and liver function parameters Alat and Gt was conducted with the Katiska software (http://www.finndiane.fi/software/katiska/) (29).

## Results

**Figure 2:**
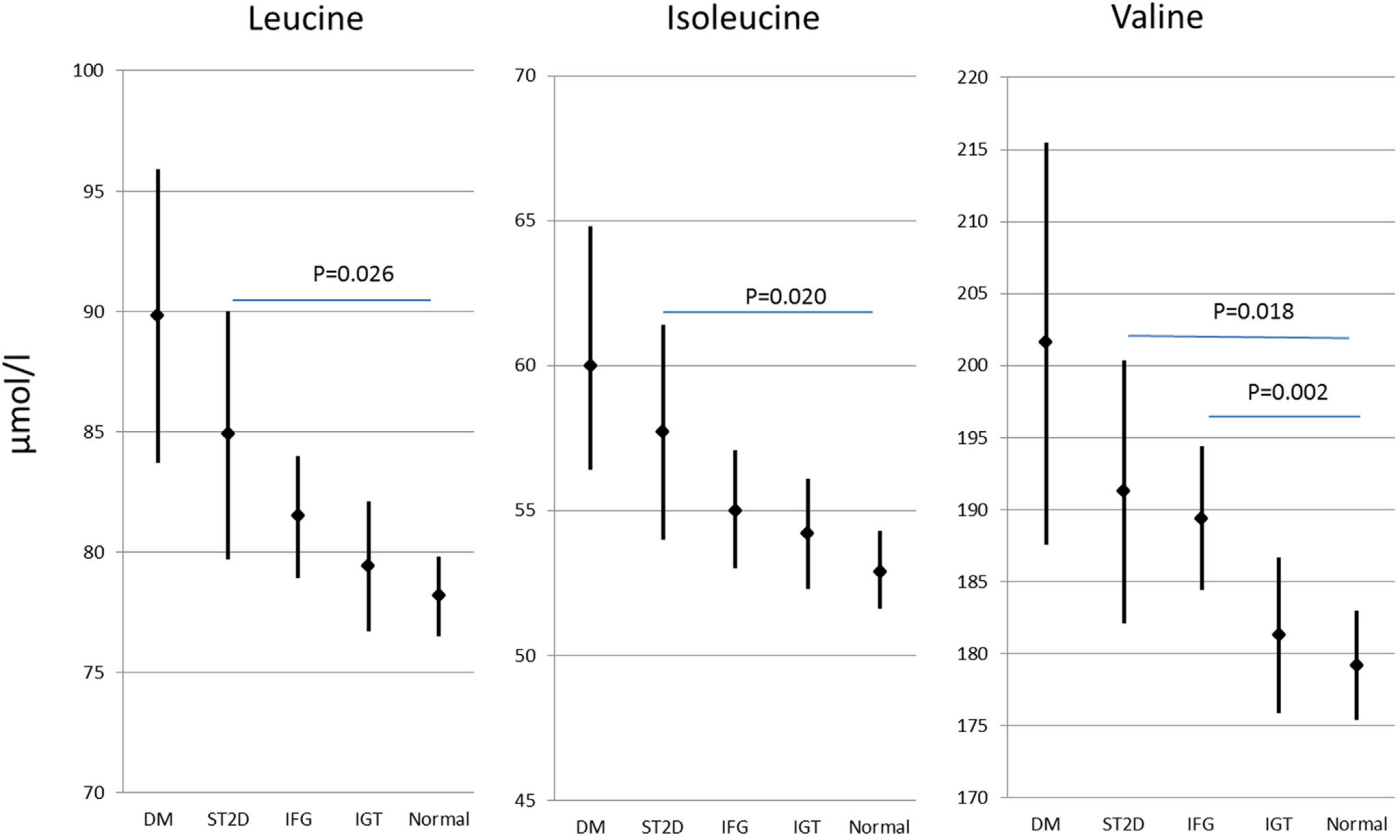
**Branched-chain amino acid concentrations (mean and 95% confidence intervals) in different diabetes/glucose level groups**. *p* values indicate difference from normal glucose group according to *post hoc* Dunn procedure. IFG, impaired fasting glucose; IGT, impaired glucose tolerance; ST2D, screen-detected type 2 diabetes; T2D, known type 2 diabetes.

Out of the 593 participants, 59 had previously known type 2 diabetes, further 67 had screen-detected type 2 diabetes (ST2D), 127 IGT, and 125 IFG, while 214 had normal fasting glucose concentration and normal glucose tolerance (data missing for OGTT for one participant). Expectedly, the isoleucine, leucine, and valine concentration correlated highly with each other (*r* ≥ 0.66 for all correlations). There were statistically significant group differences in all of the BCAA concentrations (*p* ≤ 0.005 for all BCAAs) the participants with normal glucose regulation having lowest and those with type 2 diabetes the highest BCAA concentrations. BCAA concentrations with *post hoc* statistical comparisons according to diabetes/glucose level groups are shown in Figure 2.

**Figure 3:**
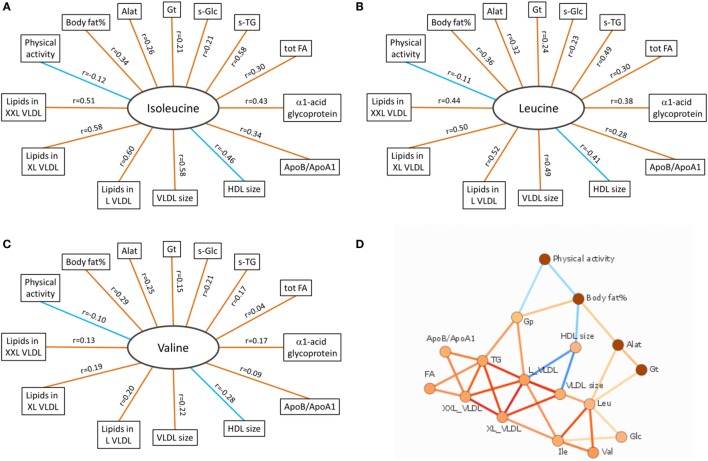
**Correlation data of BCAAs**. **(A–C)** Spearman correlations of isoleucine, leucine, and valine with other variables associated with oxidative lipid metabolism. Orange and blue lines indicate positive and negative correlations, respectively. *p* Values for all BCAAs in comparisons with body fat% were <0.0001. For physical activity (MET-h/week), the significance values were *p* = 0.003 (isoleucine), *p* = 0.008 (leucine), and *p* = 0.016 (valine). Correlations between all BCAAs and liver function analytes (Alat, γ-Gt) were highly significant (*p* < 0.0001 except between valine and γ-Gt *p* = 0.0002). Correlations between BCAAs and other selected indicators of cardiometabolic risk from metabolomics platform were highly significant (*p* < 0.0001) except between valine and tot TG (*p* = 0.28), XXL VLDL (*p* = 0.002), and ApoB/ApoA1 (*p* = 0.022). **(D)** Data-driven association network of serum BCAA concentrations and other factors related to oxidative lipid metabolism. Alat indicates alanine aminotransferase; Apo, apolipoprotein; Gp, α1-acid glycoprotein; Gt, γ-glutamyltransferase; HDL, high-density lipoprotein; L VLDL, XL VLDL, and XXL VLDL, large, very large, and extremely large very-low-density lipoprotein, respectively; s-Glc, serum glucose; s-TG, serum triglycerides; tot FA, total fatty acids. In **(A–C)**, red line denotes positive and blue line negative correlation; in **(D)**, red line denotes positive and blue line negative association, and more intensive color denotes higher association.

**Figure 4:**
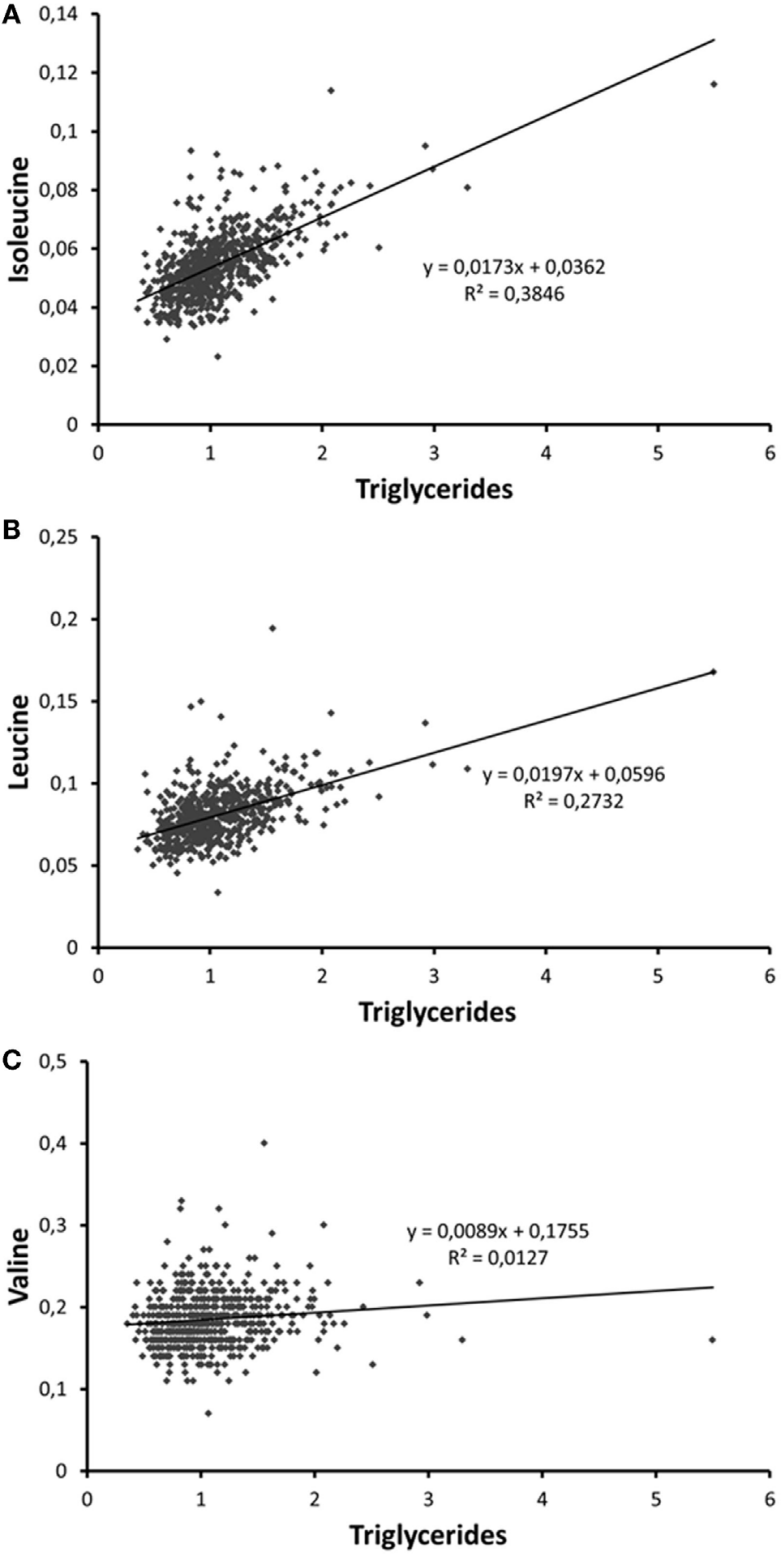
**Scatter plots depicting the associations of serum (A) isoleucine, (B) leucine, and (C) valine with serum trigyceride concentration**. The person with the highest triglyceride concentration is a patient with insulin treatment for type 2 diabetes.

According to the International Diabetes Federation criteria, 323 participants had metabolic syndrome. Those participants with metabolic syndrome had higher BCAA concentrations compared to those without (*p* < 0.001 for each of the BCAAs, Table 2) (data missing for 5). All BCAA concentrations correlated positively with body fat percentage (*r* = 0.29–0.34, *p* < 0.0001 for all). Also, all BCAA concentrations correlated with alanine aminotransferase and γ-glutamyltransferase concentration, *p* ≤ 0.0002 for all correlations (Figure 3). In addition, statistically significant negative correlations between recent leisure-time physical activity (MET-h/week) and isoleucine, leucine, and valine were observed. Associations with fasting glucose and the selected indicators of disturbed oxidative lipid metabolism/cardiometabolic risk from metabolomics are also shown in Figure 3. When adjusted with age, physical activity (MET-h/week) and body fat percentage these correlations remained at the same level (only for valine some correlations were attenuated). These correlations with 95% confidence intervals are presented in Table S1 in Supplementary Material. Expected associations with high BCAA concentrations and unfavorable metabolic profile indicators were found. Interestingly, the associations were stronger with isoleucine and leucine than with valine except for fasting glucose showing similar level of associations. Stronger association of isoleucine and leucine with serum triglycerides than that of valine was also seen (Figure 4).

**Table 2:** **BCAA values according to presence of metabolic syndrome**.

	Occurrence of metabolic syndrome		
	Yes (*n* = 323)	No (*n* = 265)	*p*-Value*

	Mean (95% CI)		
Isoleucine, μmol/L	58 (57–60)	51 (50–53)	<0.001
Leucine, μmol/L	85 (83–86)	77 (75–79)	<0.001
Valine, μmol/L	191 (188–195)	178 (175–182)	<0.001

*p Values are for difference between those with and without metabolic syndrome by Mann–Whitney U-test.

## Discussion

Our study is consistent with and extends the evidence for the hypothesis that BCAA catabolism and oxidative energy metabolism and metabolic disturbances are intertwined *via* the use of BCAAs in TCA cycle.

### Strengths and Limitations

In this study, we did not have diary-based food records to study how BCAA intake influences serum BCAA concentrations. Instead, we had food frequency questionnaire (30) based calculations of different BCAA intakes. These intakes did not correlate with fasting serum BCAA concentrations, which is in line with the idea that our findings were not due to variations in dietary intake of the studied BCAAs (9). Some of the blood lipids were analyzed both using standard clinical chemistry methods and the metabolomics platform. These measurements correlated highly and using values from these differing measurement techniques gave very similar results.

The use of BIA to measure body composition may be regarded as a limitation. BIA was used in order to provide comparable results between the study centers, since all centers did not have a possibility to use dual-energy x-ray absorptiometry (DEXA) that is considered one of the main methods to measure body composition. Another limitation is the cross-sectional nature of the study setup that does not allow causal interpretation. Furthermore, only males were included to the study so the results cannot be extrapolated to women.

### Comparison with Other Studies

Our results agree with earlier data showing that in rodents and humans with impaired glucose regulation, increased BCAA concentrations have been reported (4, 31, 32). These findings are in line with our hypothesis that serum BCAAs are elevated when the catabolism of BCAAs to be used in oxidative energy metabolism in TCA cycle is disturbed. Also, in agreement with previous data, BCAA concentrations were higher among participants with metabolic syndrome compared to those without. Expectedly, high body fat percent that is a characteristic for individuals with metabolic syndrome associated with elevated BCAA concentrations. The BCAA concentrations are increased in particular among obese individuals with metabolic syndrome (21). The findings on the association between recent high physical activity level and reduced levels of BCAAs are in agreement with recent data (9). Of the commonly used serum analytes reflecting liver function, serum alkaline phosphatase, and γ-glutamyltransferase have been shown to be most strongly related to the occurrence of non-alcoholic fatty liver disease NAFLD (20). Serum BCAA concentrations associated with these analytes. In metabolic syndrome, there is an accumulation of large VLDL and postprandial remnants lipoproteins, which are known to have high atherogenic potential (18), while large HDL particles are less atherogenic than small HDL particles (9, 16, 19). On the basis of this knowledge, we selected from the metabolomics platform a set of variables typically indicating atherogenic metabolic profile related to insufficient oxidative lipid metabolism, and found strong associations with serum BCAA concentrations, in particular with isoleucine and leucine.

The findings are in line with our BCAA–FatOx hypothesis (15), although they do not give final proof on the hypothesized mechanism. The BCAA–FatOx hypothesis (see Figure 1 for more details) proposes how BCAA degradation is mechanistically connected to lipid storage and oxidation in skeletal muscle (15). Shortly, the first reaction of the BCAA catabolism in mitochondria is the transfer of amino group from BCAA to a TCA cycle intermediate α-ketoglutarate to form glutamate by the mitochondrial branched-chain aminotransferase. Glutamate and mitochondrial oxaloacetate form aspartate that is transferred *via* malate–aspartate shuttle to cytosol. In our model, cytosolic aspartate is processed to oxaloacetate that is further metabolized to phosphoenolpyruvate for glyceroneogenesis. Glyceroneogenesis is required in skeletal muscles for the storage of fatty acids in intramyocellular lipid droplets. Similar to BCAA catabolism, β-oxidation of fatty acids from lipid droplets provides acetyl-CoA and also succinyl-CoA. Briefly, we suggest that BCAA catabolism is essential for cytosolic oxaloacetate formation and thereafter for storing fatty acids as lipid droplets. Accordingly, as in the present study, disturbed lipid metabolism can be seen as higher serum concentrations of BCAAs.

Interestingly, the associations of indicators of disturbed oxidative fat metabolism with valine were weaker than with isoleucine and leucine (see Table S1 in Supplementary Material). This may be due to the fact that valine is catabolized to succinyl-CoA, while degradation of isoleucine and leucine provides acetyl-CoA to the TCA cycle, which may be a more rate-limiting step.

### Unanswered Questions and Future Research

We also calculated correlations with the indicators of impaired lipid metabolism and other serum amino-acid concentrations measured using the metabolomics platform (alanine, glutamine, glycine, histidine, phenylalanine, and tyrosine), but only three correlations showed a moderate correlation (*r* > 0.3) with phenylalanine (results not shown). It is to note that BCAA and aromatic amino acid metabolism may have other multiple associations with different metabolites (33), but the analyses we presented only focused on the mechanistic key link between the catabolism of BCAAs and lipid metabolism.

Of the BCAAs leucine and its catabolites, α-ketoisocaproic acid and β-hydroxy-β-methylbutyrate are known activators of mTOR pathway, thus regulating muscle protein synthesis (34). Also, the intermediate product of valine catabolism, 3-hydroxyisobutyrate, has been shown to be secreted out of muscle cells and to activate in a paracrine manner transendothelial fatty acid transport and to promote muscle fatty acid uptake (35). The supplementation of the third BCAA, isoleucine, has been found to increase the expression of the proton uncoupler UCP3 and the molecular markers of lipid mobilization as well as to reduce body and adipose tissue weight gain during a high-fat diet (36). It remains to be shown what is the actual mechanism of isoleucine in concert with other BCAAs in the suggested regulation of basal metabolic rate.

## Author Contributions

Conception and design was carried out by UK and HK; all the authors were involved with the acquisition, analysis, and interpretation of data; UK and HK drafted the article, while critical revision of the manuscript was carried out by all authors. All the authors approved the final version of the paper to be published. UK is the guarantor of this work.

## Conflict of Interest Statement

The authors declare that the research was conducted in the absence of any commercial or financial relationships that could be construed as a potential conflict of interest.
