# Supplementary material for: Branched-Chain Amino Acid Levels Are Related with Surrogates of Disturbed Lipid Metabolism among Older Men
Source: Front Med (Lausanne). 2016 Nov 25;3:57. doi: 10.3389/fmed.2016.00057 (PMC5122573; doi:10.3389/fmed.2016.00057)
Supplement: Supplementary file 1 [file table_1.docx]

Supplementary Material

**Branched-Chain Amino Acid Levels Are Related with Surrogates of Disturbed Lipid Metabolism among Older Men**

**Urho M. Kujala, Markku Peltonen, Merja K. Laine, Jaakko Kaprio, Olli J. Heinonen, Jouko Sundvall, Johan G. Eriksson, Antti Jula, Seppo Sarna, Heikki Kainulainen^*^**

*** Correspondence:** Heikki Kainulainen, heikki.s.o.kainulainen@jyu.fi

**Supplementary Table 1.** Age-, physical activity- (MET-h/wk) and body fat % -adjusted correlations* with 95% CIs and *p*-values between BCAAs and other variables known to be associated with oxidative lipid metabolism.

|  | Isoleucine | Leucine | Valine |
| --- | --- | --- | --- |
| **Liver function parameters** |  |  |  |
| Alanine aminotransferase, U/l | .12 (.05‒.019)  p=.006 | .14 (.06‒.22)  p=.001 | .13 (.05‒.20)  p=.003 |
| γ-glutamyltransferase, U/l | .22 (.11‒.33)  p<.001 | .25 (.13‒.37)  p<.001 | .12 (.07‒.23)  p=.005 |
| **Selected indicators of cardio-metabolic risk from metabolomics platform** |  |  |  |
| Glucose, mmol/L | .19 (.11‒.27)  p<.001 | .26 (.15‒.39)  p<.001 | .21 (.11‒.36)  p<.001 |
| Serum total triglycerides, mmol/L | .60 (.51‒.67)  p<.001 | .48 (.37‒.59)  p<.001 | .03 (-.06‒.14)  p=.514 |
| Total fatty acids, mmol/L | .37 (.25‒.48)  <.001 | .37 (.24‒.49)  p<.001 | -.02 (-.10‒.07)  p=.731 |
| Total lipids in chylomicrons and extremely large VLDL, µmol/L | .56 (.48‒ .63)  p<.001 | .48 (.38‒.59)  p<.001 | .01 (-.09‒.14)  p=.876 |
| Total lipids in very large VLDL, µmol/L | .60 (.51‒.67)  p<.001 | .50 (.38‒.61)  p<.001 | .03 (-.06‒.14)  p=.520 |
| Total lipids in large VLDL, µmol/L | .62 (.53‒.68)  p<.001 | .49 (.38‒.60)  p<.001 | .04 (-.05‒.15)  p=.334 |
| Mean diameter for VLDL particles, nm | .56 (.47‒.63)  p<.001 | .40 (.28‒.51)  p<.001 | .08 (-.02‒.18)  p=.054 |
| Mean diameter for HDL particles, nm | -.33 (-.42‒ -.24)  p<.001 | -.25 (-.35‒ -.14)  p<.001 | -.16 (-.24‒ -.08)  p<.001 |
| Ratio of apolipoprotein B to apolipoprotein A-I | .34 (.26‒.42)  <.001 | .27 (.17‒.36)  p<.001 | .06 (-.04‒.15)  p=.189 |
| Glycoprotein acetyls, mainly α1-acid glycoprotein, mmol/L | .48 (.37‒.57)  p<.001 | .41 (.29‒.53)  p<.001 | .09 (-.01‒.19)  p=.034 |

*Correlations calculated with bootstrapping for 1000 samples
